# Antituberculosis Drugs (Rifampicin and Isoniazid) Induce Liver Injury by Regulating NLRP3 Inflammasomes

**DOI:** 10.1155/2021/8086253

**Published:** 2021-02-19

**Authors:** Qiang Su, Wei Kuang, Weiyi Hao, Jing Liang, Liang Wu, Chunmei Tang, Yali Wang, Tao Liu

**Affiliations:** ^1^Department of Pharmacy, Nanchong Central Hospital, The Second Clinical Medical College, North Sichuan Medical College, Nanchong, Sichuan, China; ^2^Nanchong Key Laboratory of Individualized Drug Therapy, Nanchong, Sichuan, China; ^3^School of Pharmacy, North Sichuan Medical College, Nanchong, Sichuan, China; ^4^Department of Cardiology, Nanchong Central Hospital, The Second Clinical Medical College, North Sichuan Medical College, Nanchong, Sichuan, China

## Abstract

Patients being treated for pulmonary tuberculosis often suffer liver injury due to the effects of anti-TB drugs, and the underlying mechanisms for those injuries need to be clarified. In this study, rats and hepatic cells were administrated isoniazid (INH) and rifampin (RIF) and then treated with NLRP3-inflammasome inhibitors (INF39 and CP-456773) or NLRP3 siRNA. Histopathological changes that occurred in liver tissue were examined by H&E staining. Additionally, the levels IL-33, IL-18, IL-1*β*, NLRP3, ASC, and cleaved-caspase 1 expression in the liver tissues were also determined. NAT2 and CYP2E1 expression were identified by QRT-PCR analysis. Finally, *in vitro* assays were performed to examine the effects of siRNA targeting NLRP3. Treatment with the antituberculosis drugs caused significant liver injuries, induced inflammatory responses and oxidative stress (OS), activated NLRP3 inflammasomes, reduced the activity of drug-metabolizing enzymes, and altered the antioxidant defense system in rats and hepatic cells. The NLRP3 inflammasome was required for INH- and RIF-induced liver injuries that were produced by inflammatory responses, OS, the antioxidant defense system, and drug-metabolizing enzymes. This study indicated that the NLRP3 inflammasome is involved in antituberculosis drug-induced liver injuries (ATLIs) and suggests NLRP3 as a potential target for attenuating the inflammation response in ATLIs.

## 1. Introduction

Tuberculosis (TB) is a disease caused by infection with *Mycobacterium tuberculosis* [1]. In recent years, extrapulmonary TB infections and atypical TB infections have become more frequently diagnosed [2], and TB remains one of the leading causes of illness and death worldwide [3]. According to a 2016 report by the World Health Organization (WHO), one-third of the world's population (~2 billion people) has been infected with TB [4]. In 2015, there were 10.4 million new cases of TB worldwide, 580,000 multidrug-resistant TB patients, and 1.4 million people died from TB [5]. At present, TB is mainly treated using anti-TB drugs [6], which can be divided into categories of new, first-line, and second-line drugs. First-line anti-TB drugs are currently the first choice for treating TB and include rifampicin (RIF), isoniazid (INH), ethambutol (E), and pyrazinamide (Z) [7–9]. However, when used in combination, these drugs produce different degrees of adverse effects [10, 11]; among which, anti-TB drug-induced liver injuries (ATLIs) are the most common and serious effect.

The WHO still regards INH and RFP to be irreplaceable first-line anti-TB drugs [12, 13]. INH inhibits the synthesis of mycolic acid, which is specific to *Mycobacterium tuberculosis* cells, and such inhibition causes the bacteria to die due to loss of acid resistance, hydrophobicity, and proliferation [13]. RIF inhibits bacterial RNA polymerase and prevents mRNA synthesis, resulting in bacteriostatic and bactericidal effects [14]. INH and RFP exert strong bactericidal and bacteriostatic effects on bacteria in both the breeding and resting stages [15]. The combined application of INH and RFP synergistically increases the killing of intracellular and extracellular tuberculosis bacilli and reduces drug resistance [16]. However, the incidence of hepatotoxicity becomes significantly increased when INH and RFP are administered in combination [17]. Although the liver injuries produced by clinical anti-TB drugs pose a serious problem, the mechanism for those injuries has not been fully elucidated.

Inflammation is a defensive response to the removal of dangerous stimuli from the body [18]. Inflammasomes, as a class of protein complexes distributed in the cytoplasm, can regulate inflammation via proinflammatory cytokines [19]. The NLRP3 inflammasome is one of the most widely studied and characterized inflammasomes [20]. When influenced by endogenous (e.g., ROS, lysosomal disruption) or exogenous (e.g., lipopolysaccharides) danger signals, NLRP3 inflammasomes become activated and induce immune and inflammatory responses [21]. NLRP3 inflammasomes play roles in a variety of diseases, such as atherosclerosis [22] and chronic glomerulosclerosis [23]. Studies have also revealed that NLRP3 inflammasomes significantly affect the development of liver diseases and that inhibition of NLRP3 inflammasomes can reduce liver inflammation [24]. However, it has not been proven whether NLRP3 inflammasomes participate in causing INH- and RFP-induced liver injuries.

We hypothesized that RIF- and INH-induced liver injuries might be ameliorated by inhibiting NLRP3 inflammasomes and that NLRP3 inflammasome inhibitors (INF39 and CP-456773) might help to protect against RIF- and INH-induced liver injuries.

## 2. Materials and Methods

### 2.1. Animals

Equal numbers of SPF grade Sprague-Dawley (SD) rats (aged 8-9 weeks; weight range, 250-350 g) were purchased from the animal experimental center of North Sichuan Medical College and fed a normal diet for 7 days in a SPF laboratory. All experiments were carried out in strict accordance with regulations concerning the management and protection of experimental animals at North Sichuan Medical College. The study protocol was approved by the Ethics Committee of SLAS (Approval No. SLAS-20200113-02).

### 2.2. Grouping and Antituberculosis Drug-Induced Hepatotoxicity (ATDH)

The SD rats were randomly assigned to a normal group (*n* = 6) or the INH+RIF group (*n* = 18), with equal numbers of males and females in each group. SD rats in the normal control group received 2 mL of normal saline solution via intragastric administration. SD rats in the INH+RIF group received INH (50 mg/kg, Novus Life Sciences Pvt. Ltd., Mumbai, India) and RIF (50 mg/kg, Novus Life Sciences Pvt. Ltd.) in a total volume of 2 mL once a day for 28 days. The rats in the INH+RIF group were also randomly assigned to an INF39 group (*n* = 6) and a CP-456773 group (*n* = 6). Samples of blood serum and liver tissue were collected from the rats in each group at 28 days after continuous drug administration. The serum was stored at -80°C; one portion of each liver sample was immersed in 4% formaldehyde, and the other portion of liver tissue was stored at -80°C for use in subsequent experiments.

### 2.3. Extraction and Culture of Hepatic Cells

After being fasted for 12 hrs, the SD rats were deeply anesthetized by intraperitoneal injection of 3% pentobarbital sodium (30 mg/kg). Calcium-free perfusion fluid and type IV collagenase solution (Sigma) were consecutively injected into the hepatic portal vein of the rats. Under aseptic conditions, the liver was carefully removed, placed in high-glucose DMEM (Procell; cat. no. PM150210), and then cut into pieces. After filtration, the liver cells were resuspended in a high-glucose DMEM and purified with Percoll reagent. After centrifugation, the pelleted liver cells were diluted with moderate low-glucose DMEM (HyClone; GE Healthcare Life Sciences, Marlborough, MA, USA) and incubated overnight in a 6-well plate. The medium was then replaced with a low-glucose DMEM containing 0.25% BSA. Albumin-conjugated oleic acid was used to induce the hepatic cells. The different groups of hepatic cells were then treated with INH and RIF, followed by treatment with INF39 or CP-456773, respectively.

### 2.4. RNA Interference

NLRP3 siRNA and a negative control (NC) were purchased from Genepharm Company (Shanghai, China). The isolated hepatic cells were transfected with NC siRNA or NLRP3 siRNAs using Lipofectamine 3000 Reagent (Invitrogen; Thermo Fisher Scientific, Inc., Waltham, MA, USA): siRNA 1 (5′-3′): GGCUAUGUACUAUCUGCUA; siRNA 2 (5′-3′): GGAUCUUUGCAGCGAUCAA; siRNA 3 (5′-3′): GGAUAGGUUUGCUGGGAUA; NC: GAGAUCUGCUUAGAUCGCA.

### 2.5. H&E Staining

The right lobe of each liver (5 mm × 5 mm × 3 mm) was fixed with 4% formaldehyde solution and embedded in paraffin. Next, tissue slices were prepared and stained with hematoxylin (Servicebio, China) for 5 mins, differentiated by exposure to hydrochloric acid alcohol solution for 20 s, and then exposed to a weak ammonia solution (Sinopharm, Ecuador, 100021600) for 20 s. After staining with eosin (Solarbio, Turkey; G1100), the slices were dehydrated and made transparent. Finally, the pathological characteristics of the liver tissues were observed under a microscope (Nikon, Japan).

### 2.6. ELISA Assay

The levels of IL-33, IL-18, and IL-1*β* were examined using an IL-33 ELISA kit (GenWay Biotech, Inc., San Diego, CA, USA), IL-18 ELISA kit (MBL, Nagoya, Japan), and IL-1*β* ELISA kit (R&D Systems, Minneapolis, MN, USA), respectively, according to instructions provided by the manufacturers. The absorbance of each sample was determined at 450 nm.

### 2.7. RNA Extraction and Real-Time Quantitative PCR (QRT-PCR) Assay

The total RNA was extracted from tissue samples and cells using Trizol reagent (Takara, Japan, cat. no. 9109). The concentration and purity of RNA were monitored by an ultraviolet detector at wavelengths of 260 nm and 280 nm, respectively. cDNA was synthesized using a reverse transcription kit (Takara) and subsequently used as a template for PCR amplification that was performed using the SYBR GREEN PCR Master Mix (Applied Biosystems, Foster City, CA, USA). The levels of mRNA expression were determined using the 2^–*ΔΔ*Ct^ method [25].

### 2.8. Western Blotting Analysis

The liver tissues in each group were ground, and the hepatic cells in each group were harvested and washed with PBS. Total proteins were extracted using a protein extraction kit (BestBio; BB-3101), and the protein concentration in each extract was determined using the bicinchoninic acid (BCA) method. Next, a 20 *μ*g aliquot of total protein from each extract was separated by 10% SDS-PAGE performed at 120 V. The separated protein bands were electrophoretically (200 mA for 90 mins) transferred onto PVDF membranes (Roche, Basal Switzerland, cat. no. 3010040001), which were subsequently blocked with 5% powdered skim milk. Next, the PVDF membranes were incubated with primary antibodies at 4°C overnight. After washing, the PVDF membranes were soaked with an HRP-labeled secondary antibody for 1 h and the immunostained protein bands were detected using the ECL chemiluminescence reagent (Millipore; KLS0500). The primary antibodies used in the study were as follows: NLRP3 (1 : 1000, Abcam, Cambridge, UK, ab214185), ASC (1 : 1000, Abcam, ab180799), and caspase 1 (1 : 1000, Abcam, ab62698).

### 2.9. Biochemistry Parameters

The liver tissues from the SD rats in each group were accurately weighed and then homogenized in normal saline. Liver tissue homogenates with a 10% mass fraction were prepared and stored at 4°C. After centrifugation at 3000g for 10 mins, the levels of superoxide dismutase (SOD), catalase (CAT), and glutathione peroxidase (GPX) activity as well as the levels of reduced glutathione (GSH) and lipid peroxidation products (LPOs) in the supernatants were determined using assay kits according to instructions provided by the manufacturers.

### 2.10. Immunohistochemistry (IHC) Assay

Immunohistochemistry was performed using the MaxVision (TM) method as previously described [26]. The tissue sections were dewaxed with xylene and then dehydrated using a gradient alcohol series. After soaking in 1% H_2_O_2_ for 10 mins, the sections were treated with citrate buffer for antigen retrieval. Next, the sections were blocked with goat serum for 1 h and treated with anti-NLRP3 (Abcam) at 4°C overnight; after which, they were incubated with a secondary antibody (Abcam) for 30 mins. Finally, the tissue sections were stained with DAB, dehydrated, and blocked. NLRP3 expression was confirmed under a microscope.

### 2.11. Immunofluorescence (IF) Assay

Treated hepatic cells were incubated for 8 hrs in a 6-well plate (5 × 10^4^ cells/well). Next, the cells were fixed in 4% paraformaldehyde (Sigma-Aldrich, St. Louis, MO, USA, cat. no. P6148-500G) for 30 mins and permeated in 0.1% Triton X100 for 10 mins. The cells were then blocked with 5% BSA for 1 h, incubated with anti-NLRP3 (Abcam) overnight at 4°C, and subsequently treated with a secondary antibody (Abcam) for 2 hrs in the dark. The cells were then stained with DAPI (Life Technologies, Carlsbad, CA, USA, cat. no. D1306,) for 10 mins, and their fluorescence was photographed under a fluorescence microscope.

### 2.12. Flow Cytometry Detection

Hepatic cells in each group were collected and counted. Next, a 200 *μ*L aliquot of suspended cells was added to 20 *μ*L of H2DCF-DA (10 *μ*mol/L; Beyotime, cat. no. S0033-1) and incubated for 15 mins in the dark. After washing with Earle's solution, the hepatic cells were suspended in 400 *μ*L of Earle's solution, and the ROS level was confirmed by flow cytometry.

### 2.13. Statistical Analysis

All experiments were independently repeated at least three times, and results are expressed as a mean value ± standard deviation (SD). Student's *t* test was used to analyze differences between two groups, and one-way analysis of variance (ANOVA) was used to evaluate the significance of differences between more than two groups. All experimental data were analyzed using IBM SPSS Statistics for Windows, Version 19.0 software (IBM Corp., Armonk, NY, USA). A *P* value < 0.05 was considered to be statistically significant.

## 3. Results

### 3.1. INH and RIF Induced Liver Injury, Enhanced the Inflammatory Response, and Activated the NLRP3 Inflammasome in Rats

**Figure 1:**
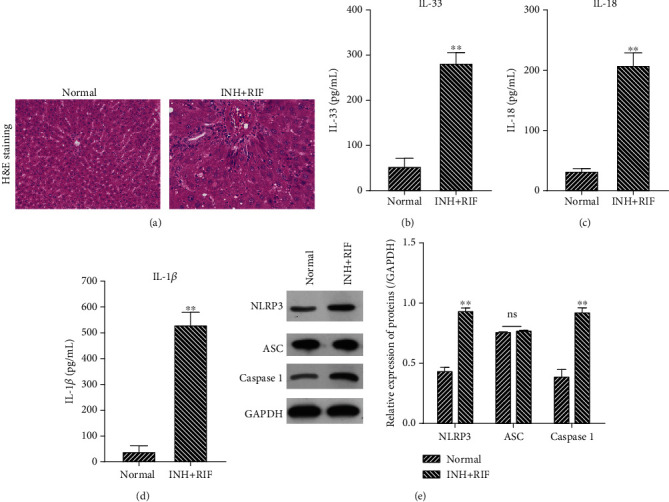
INH and RIF induced liver injury, enhanced the inflammatory response, and activated NLRP3 inflammasomes in rats. Rats were administrated 70 mg/kg INH and 70 mg/kg RIF for 28 consecutive days. (a) The liver tissues in each group were collected, and histopathological changes in the liver tissues were observed after H&E staining. Samples of blood serum were obtained from each group of rats; the levels of IL-33 (b), IL-18 (c), and IL-1*β* (d) in serum were detected by ELISA. (e) Western blot assays were performed to verify the levels of NLRP3, ASC, and cleaved-caspase 1 protein expression in each group of liver tissues. GAPDH served as an internal reference. A quantitative analysis of each protein expression was conducted according to the gray value. ^∗∗^*P* < 0.01 vs. the normal group.

In order to determine the effects of INH and RIF on the histopathological characteristics of liver tissues, an inflammatory response and NLRP3 inflammasome ATDH model was established in SD rats by dosing the rats with INH (70 mg/kg) and RIF (70 mg/kg) for 28 consecutive days. Subsequent H&E staining showed that the liver tissues from control rats had a normal morphology and intact structure, and no degeneration or necrosis was observed. In contrast, liver tissues from the rats dosed with INH and RIF contained necrotic hepatocytes and showed signs of inflammatory cell infiltration (Figure 1(a)). Moreover, the levels of inflammatory cytokines (IL-33, IL-18, and IL-1*β*) in the INH and RIF group were significantly elevated when compared with those in the normal group (*P* < 0.01, Figures 1(b)–1(d)). We also found that the levels of NLRP3 inflammasome-related proteins (NLRP3, ASC, and cleaved-caspase 1) were markedly upregulated in the INH and RIF group when compared with those in the normal group (*P* < 0.01, Figure 1(e)). When taken together, these findings indicated that the antituberculosis drugs INH and RIF could cause liver injury, induce an inflammatory response, and activate NLRP3 inflammasomes in rats.

### 3.2. INH and RIF Markedly Regulated the OS-Antioxidant Defense System and Drug-Metabolizing Enzymes in Rats

**Figure 2:**
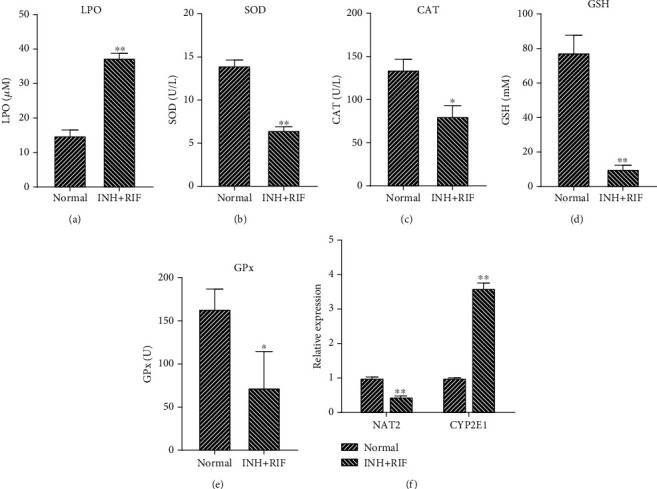
INH and RIF markedly regulated the OS-antioxidant defense system and drug-metabolizing enzymes in rats. (a–e) The OS indices (LPOs) and levels of antioxidant enzymes (SOD, CAT, GSH, and GPx) in rats treated with INH and RIF were determined using specific commercial kits. (f) QRT-PCR analyses of NAT2 and CYP2E1 in the liver tissues of rats treated with INH and RIF. ^∗^*P* < 0.05 and ^∗∗^*P* < 0.01 vs. the normal group.

As the most active metabolic organ in the body, the liver performs crucial functions, such as material metabolism, energy metabolism, and biological transformation of various molecules. In subsequent experiments, we investigated the effects of INH and RIF on liver drug-metabolizing enzymes, oxidative stress (OS), and antioxidant enzyme activity in rats. We found that the levels of OS indices (LPOs) in the INH and RIF group were significantly higher than those in the normal group (*P* < 0.01, Figure 2(a)). We also found that the levels of antioxidant enzymes (SOD, CAT, GSH, and GPx) in the INH and RIF group were significantly lower than those in the normal group (*P* < 0.01, Figures 2(b)–2(e)). Moreover, NAT2 expression was markedly downregulated and CYP2E1 expression was markedly upregulated in the INH and RIF group when compared with the normal group (*P* < 0.01, Figure 2(f)). Thus, our data revealed that INH and RIF could significantly reduce antioxidant functions and also the activity of drug-metabolizing enzymes in rat liver tissue.

### 3.3. The NLRP3 Inflammasome Was Required for the INH/RIF-Induced Inflammatory Response in Rats

**Figure 3:**
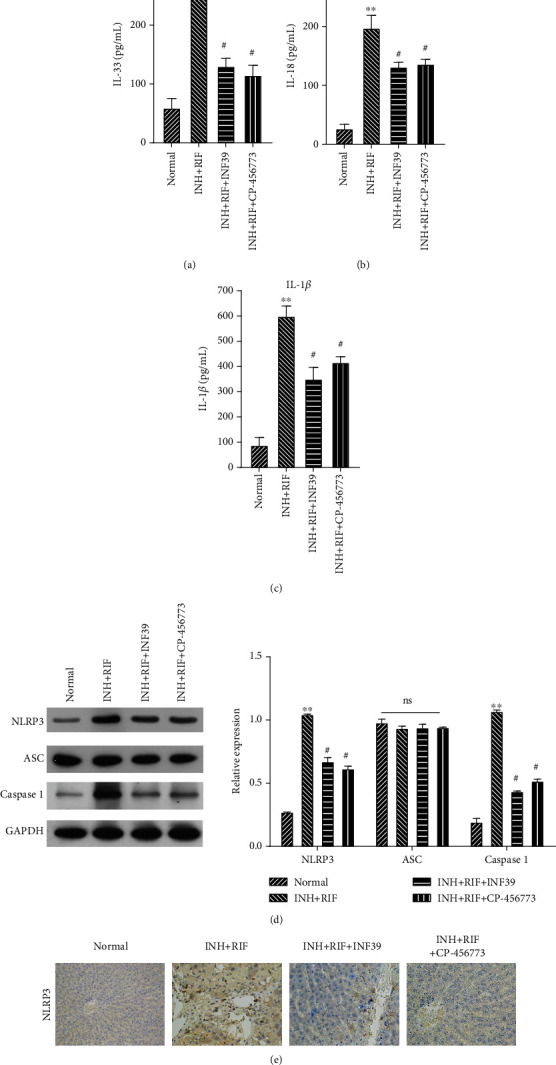
NLRP3 inflammasomes were required for INH- and RIF-induced inflammatory responses in rats. Rats were treated with INH and RIF for 28 consecutive days and then treated with an NLRP3-inflammasome inhibitor (INF39 or CP-456773, respectively). (a–c) ELISA assays were performed to evaluate the effects of NLRP3 inhibitors on INH- and RIF-induced inflammatory factors (IL-33, IL-18, and IL-1*β*) in the serum of each group of rats. (d) The levels of NLRP3, ASC, and cleaved-caspase 1 protein expression were assessed by western blot assays, and the relative levels of the proteins were analyzed based on the gray value. (e) IHC assays revealed the expression and distribution of NLRP3 in the liver tissues of rats in each group. Magnification, ×100. ^∗∗^*P* < 0.01 vs. the normal group; ^#^*P* < 0.05 vs. the INH and RIF group.

Next, we explored whether the NLRP3 inflammasome helps to facilitate the inflammatory response in INH- and RIF-induced rats by treating the rats with NLRP3-inflammasome inhibitors (INF39 or CP-45677, respectively). Subsequent ELISA assays showed that the levels of IL-33, IL-18, and IL-1*β* in rats treated with INH and RIF were significantly enhanced when compared to rats in the normal group, and rescue experiments verified that treatment with INF39 or CP-456773 could partly attenuate the INH- and RIF-mediated increases in IL-33, IL-18, and IL-1*β* levels in rat serum (*P* < 0.05 and *P* < 0.01, Figures 3(a)–3(c)). Additionally, we also found that the significant increases in NLRP3, ASC, and cleaved-caspase 1 expression in rat liver tissue caused by RIF and INH administration could be markedly reduced by an NLRP3-inflammasome inhibitor (INF39 or CP-456773) (*P* < 0.05 and *P* < 0.01, Figure 3(d)). Similarly, the results of IHC assays verified that INF39 or CP-456773 could notably weaken the promoting effect of RIF and INH on NLRP3 expression in the liver tissues of rats (Figure 3(e)). When taken together, our data showed that INH and RIF induced a strong inflammatory response in rat liver tissue by activating NLRP3 inflammasomes.

### 3.4. NLRP3 Inflammasomes Altered the INH- and RIF-Mediated OS-Antioxidant Defense System and the Levels of Drug-Metabolizing Enzymes in Rats

**Figure 4:**
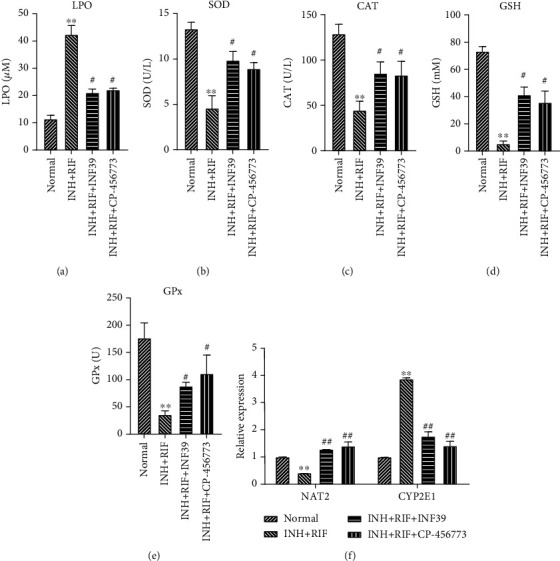
The NLRP3 inflammasome was involved in regulating the INH- and RIF-mediated OS-antioxidant defense system and drug-metabolizing enzymes in rats. INF39 or CP-456773 was administered to the INH- and RIF-induced rats, respectively. (a–e) Specific commercial kits were used to monitor the levels of LPO and antioxidant enzymes (SOD, CAT, GSH, and GPx). (f) NAT2 and CYP2E1 expression were detected by QRT-PCR assays. ^∗∗^*P* < 0.01 vs. the normal group; ^#^*P* < 0.05 and ^##^*P* < 0.01 vs. the INH and RIF group.

Likewise, we also examined the effects of NLRP3 inflammasomes on antioxidant and drug-metabolizing enzymes in rats. Our data showed that the increases in LPOs that were mediated by INH and RIF in rats could be significantly attenuated by INF39 or CP-456773 (*P* < 0.05 and *P* < 0.01, Figure 4(a)). Subsequently, we also found that either INF39 or CP-456773 could reverse the decreases in antioxidant enzyme levels (SOD, CAT, GSH, and GPx) caused by treatment with INH and RIF (*P* < 0.05 and *P* < 0.01, Figures 4(b)–4(e)). Moreover, our data also showed that the downregulation of NAT2 expression and upregulation of CYP2E1 expression in INH- and RIF-stimulated rats could also be markedly changed by INF39 or CP-456773 (*P* < 0.01, Figure 4(f)). These findings indicated that NLRP3 inflammasome inhibitors (INF39 or CP-456773) could significantly reduce INH- and RIF-induced hepatotoxicity in rats.

### 3.5. INH and RIF Regulated Drug-Metabolizing Enzymes and Induced an Inflammatory Response and OS by Activating NLRP3 Inflammasomes in Hepatic Cells

**Figure 5:**
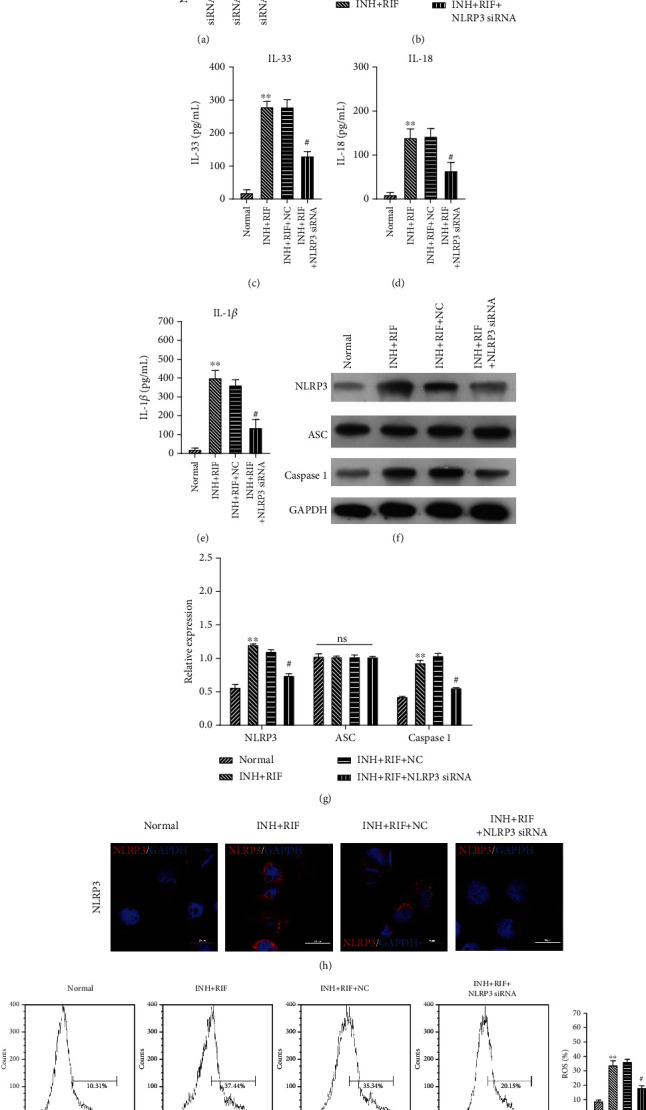
INH and RIF regulated drug-metabolizing enzymes and induced an inflammatory response and OS in hepatic cells by activating the NLRP3 inflammasome. Hepatic cells were treated with INH and RIF and then transfected with NLRP3 siRNA. (a) Transfection efficiency of siRNAs was detected by using QRT-PCR. (b) QRT-PCR analyses were performed to detect the levels of NAT2 and CYP2E1 expression in each group of hepatocytes. ELISA assays were performed to detect the levels of IL-33 (c), IL-18 (d), and IL-1*β* (e) in hepatic cells treated with INH and RIF, and INF39 or CP-456773. (f) Western blot analyses revealed changes in the levels of NLRP3, ASC. And cleaved-caspase 1 protein expression in the treated hepatic cells. (g) Protein expression was quantified based on the gray values obtained from western blotting. (h) IF assays were performed to determine the expression and distribution of NLRP3 protein in treated hepatic cells. Magnification, ×200; scale bar = 20 *μ*m. (i) ROS levels were monitored by flow cytometry. ^∗∗^*P* < 0.01 vs. the normal group; ^#^*P* < 0.05 and ^##^*P* < 0.01 vs. the INH and RIF group.

We performed *in vitro* experiments to determine whether NLRP3 inflammasomes affected drug-metabolizing enzymes, the inflammatory response, and OS in INH- and RIF-induced hepatic cells. Firstly, siRNA was transfected into cells and transfection efficiency was detected by using QRT-PCR. Results showed that siRNA 2 presented the highest efficiency of knockdown on NLRP3 (Figure 5(a)). QRT-PCR analyses showed that when compared to hepatic cells from the normal group, the levels of NAT2 were downregulated and the levels of CYP2E1 were upregulated in the INH- and RIF-induced hepatic cells, while NLRP3 knockdown dramatically reversed the levels NAT2 and CYP2E1 expression in INH- and RIF-induced hepatic cells (*P* < 0.01, Figure 5(b)). Secondly, we found that INH and RIF significantly increased the levels of IL-33, IL-18, and IL-1*β* in hepatic cells, and those increases could be significantly attenuated by NLRP3 knockdown (*P* < 0.05 and *P* < 0.01, Figures 5(c)–5(e)). Additionally, western blot studies showed that treatment with INH and RIF increased the levels of NLRP3 and cleaved-caspase 1 expression in hepatic cells, while those increases were attenuated by NLRP3 knockdown (*P* < 0.05 and *P* < 0.01, Figures 5(f) and 5(g)). Graphical results of IF assays showed the same trend in NLRP3 expression as western blotting results and also showed that NLRP3 protein was mainly located in the cytoplasm (Figure 5(h)). Furthermore, we verified that the levels of ROS were significantly elevated in hepatic cells in the INH plus RIF group when compared with hepatic cells in the normal group, and those increases could be attenuated by NLRP3 knockdown (Figure 5(i)). Therefore, we proved that INH and RIF dramatically reduced the activity of drug-metabolizing enzymes and induced an inflammatory response and OS in hepatic cells by regulating NLRP3 inflammasomes.

## 4. Discussion

Our study showed that the anti-TB drugs INH and RIF could significantly change the structure of normal liver tissues and induce inflammation. The mechanism by which anti-TB drugs cause liver injury is quite complicated [27, 28]. Current studies have indicated that the pathogenesis of ATLI involves both hepatotoxicity and metabolic specificity [29]. Anti-TB drugs are initially transported to the liver, where they are transformed into metabolites via enzymatic reactions [30]. Subsequently, the metabolites, as immunogens, bind to endogenous proteins and subsequently cause liver immune damage or hepatotoxicity [31]. The toxic metabolites of INH can lead to heterogeneous drug reactions, which are the main cause for ADLI in most heterogeneous patients [32]. RIF can induce a variety of metabolic enzymes in the liver, and those enzymes can further aggravate the toxicity of the drug to the liver [33]. RIF- and INH-induced liver injuries have been previously reported in several studies. For instance, *Tamarix gallica* leaf extract was shown to protect against RIF- and INH-induced liver injury in rats ([34]); pyrrolidine dithiocarbamate was shown to alleviate liver injuries induced by RIF and INH in rats [35]; *naringenin* was found to significantly mitigate the effects RIF- and INH-induced hepatotoxicity [36]. However, the mechanisms for these effects remain unclear.

The body's OS-antioxidant defense system can quickly remove endogenously produced ROS from the body under normal physiological conditions [37]. However, continuous external stimulation can result in excessive ROS production that causes tissue damage [38]. Drug-metabolizing enzymes are key factors that determine how drugs are metabolized in the body [39]. It has been reported that slow NAT2 acetylator genotypes and a CYP2E1 C1/C1 genotype can lead to an accumulation of toxic metabolites during the metabolism of INH in the liver [40]. It was also found that the quantities of toxic metabolites generated by breakdown of INH and RIF were significantly increased in patients with slow NAT2 acetylator genotypes [41]. Research has confirmed that RIF can induce INH hydrolase and thereby cause liver injury in patients with slow NAT2 acetylator genotypes [41]. In our study, we verified that INH and RIF markedly increased the levels of OS indices (LPOs) and reduced the levels of antioxidant enzymes, suggesting that INH and RIF could affect the OS-antioxidant defense system. We also verified that INH and RIF could downregulate NAT2 expression and upregulate CYP2E1 expression, indicating that INH and RIF could regulate the activity of drug-metabolizing enzymes.

NOD-like receptor protein 3 inflammasomes (NLRP3 inflammasomes) comprise a class of polyprotein complexes that exist in the cytoplasm [21]. Activation of NLRP3 inflammasomes can cause downstream inflammatory cascades (Dougherty et al. 2019). The NLRP3 inflammasome is composed of NLRP3, apoptosis-associated speck-like protein containing a CARD (ASC), and procaspase 1 [42]. When stimulated by exogenous pathogens such as bacteria, viruses, and fungi, or by endogenous stimuli, NLRP3 activates caspase 1 by recruiting the adaptor protein ASC to bind to procaspase 1 [43]. Activated caspase 1 then causes activation of pro-interleukin-1*β* (pro-IL-1*β*) and pro-interleukin-18 (pro-IL-18) to form IL-1*β* and IL-18 [42, 44]. Numerous studies have verified that NLRP3 inflammasomes are involved in regulating liver injuries [45, 46]. In our study, we verified that INH and RIF could activate NLRP3 inflammasomes in the liver tissues of rats and hepatocytes cultured *in vitro*. We also demonstrated that an NLRP3-inflammasome inhibitor (INF39 or CP-456773) could markedly reverse the regulatory effects of INH and RIF on drug-metabolizing enzymes, the OS-antioxidant defense system, and inflammatory response in rats. Additionally, we showed that silencing of NLRP3 also could lessen the influence of INH and RIF on hepatic cells. Therefore, we proved that the NLRP3 inflammasome is required for INH- and RIF-induced liver injuries.

## 5. Conclusions

Our findings suggest that INH and RIF can destroy the normal liver tissue, induce an inflammatory response and OS, and also regulate drug-metabolizing enzymes and the antioxidant defense system by accelerating the activation of NLRP3 inflammasomes. Therefore, NLRP3 inflammasomes might be the key factors involved in INH- and RIF-induced liver injuries.

## Data Availability

The datasets used and/or analyzed during the present study are available from the corresponding author on reasonable request.
